# Complete mitochondrial genome of the apple snail *Pomacea diffusa* (Gastropoda, Ampullariidae) with phylogenetic consideration

**DOI:** 10.1080/23802359.2017.1407683

**Published:** 2017-11-26

**Authors:** Jing Guo, Huirong Yang, Chunxia Zhang, Huayi Xue, Yu Xia, Jia-En Zhang

**Affiliations:** a College of Natural Resources and Environment, South China Agricultural University, Guangzhou, China;; b College of Marine Sciences, South China Agricultural University, Guangzhou, China;; c Key Laboratory of Agro-Environment in the Tropics, Ministry of Agriculture, Guangzhou, China;; d Guangdong Provincial Engineering Technology Research Center of Modern Eco-agriculture and Circular Agriculture, Guangzhou, China

**Keywords:** *Pomacea diffusa*, mitochondrial genome, NGS technique, Gastropoda

## Abstract

We present the complete mitochondrial genome of *Pomacea diffusa* in this study. The results show that the mitochondrial genome is 16,640 bp in length, which is comprised of 13 protein-coding genes, two rRNA genes, and 21 tRNA genes. The nucleotide compositions of the light strand are 39.62% of A, 30.13% of T, 16.02% of C, and 14.24% of G. Except eight tRNA (Glu, Gly, Trp, Cys, Tyr, Met, Thr, Val) on the light strand, the rest are encoded on the heavy strand. All the protein-coding genes start with ATC initiation codon, and two types of inferred termination codons are TAA and TAG. There are 26 intergenic spacers and two gene overlaps. The phylogenetic analysis shows that *P. diffusa* clusters with *P. canaliculata* and *P. maculata* with high bootstrap support, which is consistent with the morphological and molecular evidence.

The apple snail *Pomacea diffusa* (Blume 1957) originated in South America and was introduced to Oceania, North America and Asia with aquarium trade (Hayes et al. 2008; Horgan et al. 2014). Unlike its congeneric invasive species *P. canaliculata* (Lamarck 1822) and *P. maculata* (Perry 1810), *P. diffusa* feeds mostly on algae and has little direct impact on macrophytes (Morrison and Hay 2011). But the hybrids between *P. diffusa* and other *Pomacea* species might pose serious threats to paddy plants and gastropod fauna (Aditya and Raut 2001a, 2001b; Morrison and Hay 2011).

Considering that *P. diffusa* was often confused with *P. bridgesii* (Reeve 1856) in the morphological identification (Hayes et al. 2008; Horgan et al. 2014) and few studies focused on *P. diffusa*, we sequenced the complete mitochondrial genome of *P. diffusa* by using the next-generation sequencing (NGS) techniques for its population genetics and polymorphism studies, which also helps to understand its introducing process and seek effective management strategy. The specimen was purchased from Yuehe Pet Market in Guangzhou (23°09′ N, 113°24′ E) and confirmed as *P. diffusa* based on morphological characteristics and molecular identification (Rawlings et al. 2007; Hayes et al. 2008). The pleopod muscle of *P. diffusa* was preserved in 95% ethanol and stored at –40 °C in South China Agricultural University, Guangzhou, China. The procedure referred from Green and Sambrook (2012) was carried out in the total genomic DNA extraction.

The complete mitochondrial genome of *P. diffusa* presented in this study (GenBank accession number KY008698) is 16,640 bp in length, containing 13 protein-coding genes, two ribosomal RNA genes (L-rRNA and S-rRNA), 21 transfer RNA genes (tRNA). All of them are encoded on the heavy strand except eight tRNA genes (Glu, Gly, Trp, Cys, Tyr, Met, Thr, Val). Among 13 protein-coding genes (total 11,268 bp) encoding 3743 amino acids, the maximum is ND5 with 1728 bp and the minimum is ATP8 with only 159 bp. The S-rRNA and L-rRNA genes are 950 and 1345 bp, respectively, located between the tRNA^Glu^ and tRNA^Leu^ genes and separated by the tRNA^Val^ gene. The inner ring indicates the GC percent varying from 22.90% to 37.60%. The overall nucleotide compositions of the light strand in descending order are 39.62% of A, 30.13% of T, 16.02% of C, and 14.24% of G. The most representative base is A, and the bias against G is observed. The absence of D-loop is consistent with the Gastropoda (Zeng et al. 2015; Yang et al. 2016a, 2016b, 2016c), but, at least, one lengthy non-coding region is an essential regulatory element for the initiation of transcription and replication (Wolstenholme 1992).

All the protein-coding genes start with ATC initiation codon, and two types of inferred termination codons are TAA (ATP8, ATP6, ND1, ND6, ND4L, ND4, ND5, COX3, ND3, ND2) and TAG (COX1, COX2, CYTB). Twenty-one tRNA genes vary from 62 to 72 bp in length, and all fold into the typical cloverleaf secondary structure. There are 26 intergenic spacers (total 1684 bp) varying from 1 to 1073 bp in length and two gene overlaps (total 27 bp), the larger of which is 20 bp between the ND5 and tRNA^Phe^ genes. The tandem repeat sequences are observed in inter genetic space of tRNA^Phe^ (GAA) and COX3 genes.

A phylogenetic tree is constructed using the maximum-likelihood method based on the complete mitochondrial genomes of the closely related 17 Gastropoda species to assess their actual phylogenetic relationship and evolution (Figure 1). *P. diffusa* clusters with *P. canaliculata* and *P. maculata* with high bootstrap support. The phylogenetic analysis is consistent with morphological and molecular evidence, indicating that *P. diffusa* has higher homology with *P. canaliculata* and *P. maculata* (80.08% and 80.45% nucleotide sequence identity, respectively) than other snail species (Figure 1; Rawlings et al. 2007; Hayes et al. 2008).

**Figure 1.**
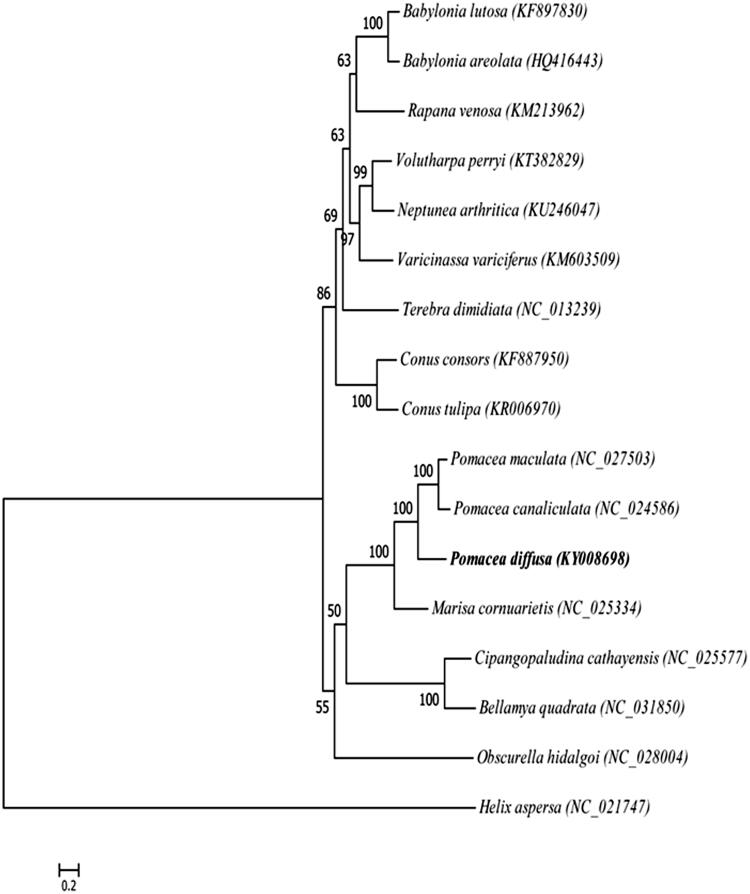
Phylogenetic tree of maximum-likelihood was constructed in RAxML based on the nucleotide sequences of 13 protein-coding genes. The bootstrap values were based on 1000 resamplings.
